# Inflammation and behavioral symptoms in preoperational glioma patients: Is depression, anxiety, and cognitive impairment related to markers of systemic inflammation?

**DOI:** 10.1002/brb3.1771

**Published:** 2020-08-13

**Authors:** Li Song, Xingyun Quan, Lin Su, Ke Wang, Haorun Wang, Lihong Wu, Chaoyi Chen, Shenjie Li, Wei Xiang, Ligang Chen, Jie Zhou

**Affiliations:** ^1^ Department of Neurosurgery the Affiliated Hospital of Southwest Medical University Lu Zhou China; ^2^ Southwest Medical University Lu Zhou China; ^3^ Sichuan Clinical Research Center for Neurosurgery Lu Zhou China; ^4^ Academician (Expert) Workstation of Sichuan Province Lu Zhou China; ^5^ Neurological Diseases and Brain Function Laboratory Lu Zhou China; ^6^ Affiliated Hospital of Traditional Chinese Medicine of Southwest Medical University Lu Zhou China

**Keywords:** anxiety symptoms, behavioral symptoms, cytokines, depression symptoms, glioma, inflammatory factor

## Abstract

**Purpose:**

Behavioral symptoms, including depression, anxiety, and cognitive impairment, are common clinical symptoms of patients with glioma. However, the mechanisms underlying the behavioral symptoms of glioma patients remain unclear. In this study, we explore the correlation between markers of systemic inflammation and preoperational behavioral symptoms in glioma patients.

**Patients and Methods:**

Patients (*n* = 71) who had recently undertaken imaging (i.e., CT, MRI) for suspected glioma had a face‐to‐face interview, completed self‐report scales, and provided blood samples. Furthermore, we tested blood samples by a protein chip to select differential inflammatory cytokines and further confirm such differences using liquid‐phase chip technology.

**Results:**

The prevalence of depression, anxiety, and cognitive impairment in glioma patients prior to surgery in this study was 53.5%, 70.4%, and 32.4%, respectively. The increased levels of IFN‐γ were positively correlated with clinical symptoms of depression in the glioma patients. Moreover, increased IL‐2 levels were negatively associated with anxiety symptoms (*p* = .00) and positively correlated with cognitive impairment in glioma patients.

**Conclusion:**

This study suggests that systemic inflammation is associated with behavioral symptoms in glioma patients. This provides further evidence of the contribution of inflammatory markers to psychological symptoms in the context of physical conditions and lays the foundation for the development of further treatments of the behavioral symptoms in glioma patients.

## Graphical abstract

The incidence of behavioral symptoms was not described before surgery in glioma patients. The correlation between behavioral symptoms and inflammation has not been studied in glioma patients before surgery.

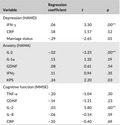

## 1 INTRODUCTION

Behavioral symptoms, including depression, anxiety, and cognitive impairment, are among the most common adverse effects of cancer diagnoses and treatment thereof (Bower, 2008).

The prevalence of depression, anxiety, and cognitive impairment has been carefully studied in patients with glioma (D'Angelo et al., 2008; Rooney, Brown, Reijneveld, & Grant, 2014; Tucha, Smely, Preier, & Lange, 2000), revealing a prevalence ranging from 6% to 93% (Loon et al., 2015; Qi et al., 2018; Zwinkels et al., 2016). However, the mechanisms underlying the behavioral symptoms of glioma patients remain unclear. Recent studies have demonstrated that behavioral changes may occur due to the metabolic changes of neurotransmitters linked to inflammatory cytokine signals, including serotonin, norepinephrine, and dopamine (Felger & Treadway, 2017). Neurotransmitters are currently the main targets for the psychopharmacological treatment of depression, anxiety, and cognitive impairment (Miller, Haroon, Raison, & Felger, 2013). However, the role of inflammatory cytokines in glioma‐related behavioral symptoms remains unclear.

The objectives of this study are the following: (a) to characterize the prevalence of depression, anxiety, and cognitive impairment in glioma patients; (b) to determine the role of tumor‐related factors on behavioral symptoms and inflammatory markers in glioma patients; and (c) to explore the correlation between influencing factors (especially inflammatory factors) and preoperational behavioral symptoms in glioma patients.

## 2 PATIENTS AND METHODS

### 2.1 Study population

This study's participants were enrolled from the Department of Neurosurgery of the Affiliated Hospital of Southwest Medical University between January 2018 and December 2019 if their imaging results indicated suspected glioma. Inclusion criteria for participants who were ultimately enrolled were as follows: (a) Patients were older than 15 years, (b) patients had received surgical treatment and pathological examination supporting the presence of glioma and were not accompanied by other underlying diseases, (c) patients spoke Chinese, (d) patients volunteered to participate in the research, and (e) patients had not received any treatment for gliomas, including drug therapy and surgery, prior to participating in the study. The exclusion criteria were as follows: (a) Patient was receiving palliative treatment, (b) patient had clinically severe dysphasia, cognitive impairment, learning disability, blind, and accompanying other diseases, (c) patient used antibiotics and psychotropic drugs, (d) patient had a medical or family history of psychological distress or mood disorder, (e) patient had a history of infection and fever in the past 2 weeks, (f) the advice of a clinician that the patient's condition was too serious to endure clinical face‐to‐face interviews, and (g) patient refused to participate in this study. The research was approved by the institutional review board of the Affiliated Hospital of Southwest Medical University, and informed consent was obtained from all participants (YX2019‐03‐02).

### 2.2 Patient demographic and medical characteristics

Demographic and clinical information was determined from self‐report questionnaires and patients’ medical records, and variables such as age, ethnicity, sex, marital status, education level, income status, epilepsy diagnosis, body mass index, Karnofsky Performance Score (KPS), course of the disease, side of the lesion, position of tumor, lump size, degree of edema (Wenlong et al., 2018), and pathological grading were included.

### 2.3 Measurement of behavioral symptoms

Participants were scheduled to receive face‐to‐face interviews with two experienced testers in order to evaluate their behavioral symptom scale and complete the self‐report scale in the communication room of the Department of Neurosurgery 2 days preoperatively. If the two testers had different opinions on the score of the participants' behavioral symptom scale, the third tester re‐evaluated the participant independently, and a consensus on the final score was achieved by the three testers. Depression was assessed using the Hamilton Depression Scale (HAMD). A score under 8 is defined as no depression; 8–20 indicates the patient “might have depression”; 21–35 indicates that there “must be depression”; and greater than 35 indicates “major depression” (Hamilton, 1960). Anxiety was assessed using the Hamilton Anxiety Scale (HAM‐A). Anxiety was scored as follows: 21–29 points—“definitely significant anxiety”; 14–21 points—“definitely anxiety”; 7–14 points—“possible anxiety”; and <7—“no symptoms of anxiety” (Hamilton, 1959). Cognitive function was assessed using the Mini‐Mental State Examination (MMSE), a well‐validated screening test for dementia and cognitive impairment for patients with gliomas (Brown et al., 2004; Gorlia et al., 2008), in which the symptoms of cognitive impairment were defined as: ≤24—“junior high school education and above,” ≤20—“primary school education,” and ≤17—“illiteracy respectively” (Folstein, Folstein, & McHugh, 1975). Functional status was measured using the KPS (Schag, Heinrich, & Ganz, 1984).

### 2.4 Inflammatory markers

On the same day, in the morning prior to the questionnaire assessment, participating patients provided biological samples (before 8 a.m.). The blood samples were collected by venipuncture into EDTA tubes, placed in a 4°C refrigerator, centrifuged for plasma isolation and acquisition, and stored at −80°C for subsequent batch inflammatory markers testing.

Liquid‐phase chip technology was used to test the specific content of each target cytokine in each sample. The levels of soluble tumor necrosis factor alpha (TNF‐α), interleukin‐1 alpha (IL‐1α), interleukin‐2 (IL‐2), interleukin‐6 (IL‐6), interleukin‐8 (IL‐8), interleukin‐10 (IL‐10), C‐reactive protein (CRP), glial cell line‐derived neurotrophic factor (GDNF), and interferon‐γ (IFN‐γ) in plasma were measured using a magnetic cytometric bead assay (LUMINEX001, EMD‐Millipore) according to the manufacturer's instructions with a Bio‐Plex‐200 system (Bio‐Rad). Initially, a volume of 50 μl of standard or sample was added per well. Next, 50 μl of diluted microparticle cocktail was added to each well and incubated for 2 hr at room temperature on a shaker at 800 rpm. Wells were washed by removing the liquid from each well, filling with 100 μl of wash buffer, and removing the liquid again. Washes were performed three times. Next, 50 μl of diluted biotin antibody cocktail was added to each well. Samples were covered and incubated for 1 hr at RT on the shaker at 800 rpm. Washing was repeated, and 50 μl of diluted streptavidin–PE was added to each well. The wash was repeated, and 100 μl of wash buffer was added to each well. The samples were incubated for 2 min at room temperature on the shaker at 800 rpm. Lastly, values were read within 90 min using a Luminex^®^ or Bio‐Rad analyzer. All samples were run in duplicate, and mean levels were used in all analyses. All analyses were measured according to the manufacturer's protocol.

### 2.5 Statistical analysis

Continuous variables were analyzed with a Mann–Whitney *U* test or Student's *t* test, and categorical variables were analyzed with a chi‐square test. Spearman's correlations were used to assess associations between behavioral symptoms. The effect of tumor‐related characteristics on symptoms and inflammatory markers was tested by a one‐way analysis of covariance (ANCOVA) (pathological grade, side of the lesion, and position of tumor) followed by Bonferroni‐corrected *t* tests for any significant outcomes. Further, univariate association between patient demographics and depression (HAMD), anxiety (HAM‐A), cognitive function (MMSE), and medical characteristics of inflammation factor was assessed using Spearman's correlation coefficient (age, body mass index, disease course, tumor area, edema area), independent *t* test (gender, grade, epilepsy, KPS, marital status), and a multigroup analysis of variance for a Bonferroni correction (category variables of lobe of glioma, laterality of glioma, level of education, level of income). Next, the covariates, which were found statistically significant in the univariate analysis, were used in the stepwise linear multivariation regression. Statistical procedures were performed using SPSS version 22 software (SPSS), and all statistical tests were 2‐tailed with a 5% significance level.

## 3 RESULTS

### 3.1 Demographic characteristics and prevalence of behavioral symptoms in glioma patients

At the beginning of the study, 143 new patients whom an imaging doctor and two clinical doctors suspected of having glioma were submitted to the research team. Altogether, 71 glioma patients were finally enrolled in this study (71/143, 49.7%) (Figure S1).

The average age of the enrolled patients was 45.5 years, and the majority were married (83.1%). Regarding the glioma grade, 33 patients had low‐grade (46.5%) and 38 had high‐grade glioma (53.5%) (Table 1). Thirty‐eight participants (53.5%) had clinically significant symptoms of depression (HAMD ≥ 8). Fifty participants (70.4%) had clinically significant symptoms of anxiety (HAM‐A ≥ 7). Twenty‐three participants had clinically significant symptoms of cognitive impairment (32.4%). Depression symptoms had an average of 10.3 points, including a female average of 11.9 points, and a male average score of 9.1 points. The average anxiety score was 10.7, with a female mean of 10.0 and a male mean of 11.3. The average cognitive score was 25.4, with a female mean of 24.6 and a male mean of 26.1 (Table S1).

**TABLE 1:** Demographic and clinical characteristics of study participants

Characteristic	Patients	
	No.	%
Age, years		
Mean	45.52	
Range	16–73	
Patient sex		
Male	40	56.3
Female	31	43.7
Married/committed relationship	59	83.1
Education status		
Low	38	53.5
Medium	31	43.7
High	2	2.8
Income		
Low	38	53.5
Medium	31	43.7
High	2	2.8
WHO glioma grade		
High grade (3–4)	38	53.5
Low grade (1–2)	33	46.5
Laterality		
Right	37	52.1
Left	29	40.8
Both	5	7.0
Lobe		
Frontal	33	46.5
Temporal	23	32.4
Other	15	21.1
Epilepsy	12	16.9
BMI		
<18.5	12	16.9
18.5–23	47	66.2
>23	12	16.9
KPS		
≥70	60	83.1
<70	11	15.5

Note

Education status: low mean primary school and below; medium mean junior high school and vocational middle school; high mean university and above. Income: low mean 1,000 RMB and below; medium mean 1,000–5,000 RMB; high mean 5,000 RMB and above it.

Abbreviations: BMI, body mass index; KPS, Karnofsky Performance Score.

### 3.2 The association of tumor‐related characteristics with behavioral symptoms and inflammatory factors

The pathological glioma grade had no significant relationship with the behavioral symptoms or inflammatory characteristics after controlling for age and sex (both *p* > .1). The other tumor‐related characteristics (side of the lesion, position of tumor, lump size, degree of edema) had no correlation with either the behavioral symptoms or inflammatory factors (Table 2).

**TABLE 2:** Differences in behavioral and inflammatory outcomes by tumor‐related characteristics

Characteristic	HAMD	HAMA	MMSE	TNF‐α	IL‐1α	IL‐2	IL‐6	IL‐8	IL‐10	CRP	GDNF	IFN‐γ
Pathological grade												
Low grade	9.05 ± 7.30	11.30 ± 8.06	26.08 ± 4.28	5.09 ± 1.56	6.64 ± 6.11	18.00 ± 7.94	3.50 ± 2.50	6.74 ± 5.05	2.73 ± 1.23	550,572.43 ± 4.11	4.65 ± 0.89	25.34 ± 3.74
High grade	11.94 ± 9.26	10.03 ± 6.36	24.61 ± 4.45	5.10 ± 1.44	8.97 ± 13.69	21.00 ± 9.05	2.76 ± 1.01	12.02 ± 16.86	2.79 ± 1.27	429,666.97 ± 4.25	4.75 ± 0.91	25.56 ± 2.95
*p*	.15	.47	.17	.98	.34	.14	.12	.07	.82	.23	.63	.80
Side of the lesion												
Left	9.62 ± 8.93	10.48 ± 6.84	24.83 ± 5.80	5.33 ± 1.62	9.21 ± 14.14	19.75 ± 9.03	2.69 ± 0.63	6.99 ± 4.83	2.83 ± 1.13	465,658.66 ± 4.18	4.69 ± 0.97	25.05 ± 2.37
Right	11.00 ± 7.92	11.32 ± 7.66	25.59 ± 3.12	4.83 ± 1.41	6.61 ± 6.36	18.37 ± 8.17	3.20 ± 1.30	10.45 ± 15.63	2.68 ± 1.31	556,900.57 ± 4.16	4.72 ± 0.89	25.48 ± 3.84
Both	9.20 ± 8.23	8.00 ± 8.33	27.80 ± 1.64	5.63 ± 1.24	6.37 ± 0.39	23.77 ± 7.91	4.82 ± 6.52	10.49 ± 9.47	2.81 ± 1.57	246,630.20 ± 4.02	4.54 ± 0.51	27.33 ± 4.92
*p*	.77	.62	.36	.29	.57	.39	.14	.49	.89	.26	.92	.39
Position of tumor												
Frontal	11.85 ± 8.56	11.52 ± 6.35	25.21 ± 4.46	5.11 ± 1.50	9.84 ± 14.59	20.12 ± 9.01	3.29 ± 2.79	8.74 ± 7.64	2.66 ± 1.30	472,999.15 ± 4.33	4.89 ± 1.22	25.51 ± 2.64
Temporal	9.57 ± 7.17	11.57 ± 8.58	24.74 ± 5.30	4.93 ± 1.57	5.79 ± 1.53	16.82 ± 7.57	3.30 ± 1.11	6.52 ± 4.71	2.82 ± 1.03	617,858.43 ± 3.94	4.45 ± 0.39	26.03 ± 4.43
Other	8.07 ± 9.09	7.80 ± 6.96	27.00 ± 1.65	5.30 ± 1.46	5.72 ± 0.95	21.36 ± 8.39	2.76 ± 0.64	13.57 ± 22.68	2.86 ± 1.46	368,190.47 ± 4.00	4.65 ± 0.40	24.35 ± 2.98
*p*	.30	.22	.28	.76	.24	.21	.67	.20	.83	.18	.19	.32
Lump size												
*r*	−0.22	−0.22	0.03	0.01	0.02	−0.01	−0.05	−0.12	0.02	0.02	−0.05	−0.05
*p*	.07	.66	.84	.95	.85	.95	.67	.34	.85	.89	.67	.67
Degree of edema												
*r*	0.04	0.14	−0.08	0.02	−0.11	−0.05	−0.08	−0.06	0.16	0.05	0.13	0.02
*p*	.72	.24	.49	.86	.35	.67	.53	.61	.17	.65	.28	.87

### 3.3 Association between influencing factors and behavioral symptoms

Marital status correlated with the HAMD scores of glioma patients (*p* < .00). Alternatively, the score of the depression symptoms of married glioma patients was significantly lower than that of unmarried glioma patients. The levels of CRP and IFN‐γ correlated positively with the HAMD score (*p* < .01; *p* < .00). People with higher KPS during hospitalization were more likely to be anxious (*p* < .00). A negative correlation was noted between the levels of IL‐1 α, IL‐2, and GDNF and the HAM‐A score (*p* < .04; *p* < .00; *p* < .04).The scores of the concentrations of TNF‐α and IL‐2, IL‐8, and GDNF correlated positively with cognitive impairment (*p* < .00, *p* < .00, *p* < .00, *p* < .01), and the level of CRP correlated negatively with cognitive impairment (*p* < .00). Depression was significantly associated with anxiety (*r* = .45; *p* < .00) (Table 3).

**TABLE 3:** Univariate analyses of demographic, inflammatory markers, and physiologic related to behavioral symptoms as measured using the HAMD, HAM‐A, and MMSE

Variable	Univariate analysis					
	Depression		Anxiety		Cognition	
	*r*	*p*	*r*	*p*	*r*	*p*
Age	0.21	.08	0.08	.53	0.00	.99
BMI	0.02	.88	−0.15	.21	0.37	.12
Time	−0.05	.67	−0.11	.35	−0.06	.60
Tumor area	−0.22	.07	−0.22	.66	−0.03	.84
Edema area	0.04	.72	0.14	.24	−0.08	.49
HAMD	—	—	0.45	.00**	−0.08	.51
HAM‐A	0.45	.00**	—	—	−0.08	.51
MMSE	−0.08	.51	−0.08	.51	—	—
TNF‐α	−0.12	.31	−0.34	.08	0.43	.00**
IL−1α	−0.02	.90	−0.25	.04*	−0.06	.61
IL−2	−0.18	.14	−0.35	.00**	0.56	.00**
IL−6	0.08	.52	0.03	.84	−0.16	.18
IL−8	−0.05	.66	−0.15	.21	0.38	.00**
IL−10	−0.07	.57	−0.07	.57	−0.12	.34
CRP	0.30	.01**	0.22	.07	−0.51	.00**
GDNF	−0.09	.49	−0.25	.04*	0.32	.01**
IFN‐γ	0.40	.00**	0.19	.12	−0.06	.63

	*t*	*p*	*t*	*p*	*t*	*p*
Gender	−1.15	.25	0.43	.69	−1.49	.14
Grade	−0.16	.84	−1.15	.25	−0.35	.73
Epilepsy	−0.27	.79	−1.21	.24	−0.75	.46
KPS	−1.47	.16	−5.64	.00*	−1.00	.32
Marrige status	3.43	.00*	1.71	.09	1.47	.16

	*F*	*p* ^#^	*F*	*p* ^#^	*F*	*p* ^#^
Lobe	1.94	.15	2.75	.07	0.67	.51
Laterality	0.73	.49	1.86	.16	0.43	.65
Education	0.96	.39	1.35	.27	3.17	.05
Income	2.84	.07	3.62	.03	0.85	.43

Note

*p* ^#^: *p* value passes through Bonferroni correction.

^*^difference is statistically significant.

^**^difference is highly statistically significant.

Next, the variables related to the demographic sociological characteristics or biological variables in the univariate analysis were included in the multiple regression analysis for each symptom. Only IFN‐γ correlated positively with depressive symptoms (*p* = .00). IL‐2 was negatively associated with anxious symptoms (*p* = .00), and IL‐2 was also significantly positively correlated with cognitive impairment (*p* = .00) (Table 4).

**TABLE 4:** Multivariate regression of significant demographic, inflammatory markers, and physiologic related to behavioral symptoms as measured using the HAMD, HAM‐A, and MMSE

Variable	Regression coefficient	*t*	*p*
Depression (HAMD)			
IFN‐γ	.06	3.30	.00**
CRP	.18	1.57	.12
Marriage status	−.29	−2.65	.01
Anxiety (HAMA)			
IL‐2	−.02	−3.25	.00**
IL‐1α	.15	1.32	.19
GDNF	.08	0.61	.54
IFNγ	.11	0.94	.35
KPS	.24	2.20	.03
Cognitive function (MMSE)			
TNF‐α	−.20	−1.04	.30
GDNF	−.14	−1.21	.23
IL−2	.03	5.80	.00**
IL−8	−.06	−0.54	.59
CRP	−.10	−0.40	.69

Note

*F* (depression) = 10.88, *p* = .00, adjusted *R* ^2^ = .12; *F* (anxiety) = 10.53, *p* = .00, adjusted *R* ^2^ = .12; *F* (cognitive function) = 33.60, *p* = .00, adjusted *R* ^2^ = .32.

^**^difference is highly statistically significant.

## 4 DISCUSSION

Depression, anxiety, and cognitive impairment were common behavioral symptoms in preoperative glioma patients. In this study, the prevalence rates of depression, anxiety, and cognitive impairment in glioma patients were 53.5%, 70.4%, and 32.4%, respectively. In glioma patients, the prevalence of depression symptoms was found to be 45.5% by BDI measurement (Jiao et al., 2015). Our results yielded rates similar to or higher than the prevalence rates of depression symptoms found in glioma patients by other investigators. Further, the prevalence of anxiety was higher than the 30%–63% reported by Rooney, Carson, and Grant (2011). This difference may be due to the different scales used to evaluate depression and anxiety symptoms and the different areas included in the population. The results of the prevalence of cognitive impairment are consistent with a systematic review of the prevalence of cognitive impairment in glioma patients published in 2015, which found an prevalence rate of 19%–83% (Loon et al., 2015).

Furthermore, the results of this study showed that tumor‐related factors, including pathological grade, side of the lesion, position of the tumor, lump size, and degree of edema, had no effect on the behavioral symptom scores or inflammatory factors. In the Moreale study, depression and anxiety symptoms in patients with gliomas were, similarly, not found to be associated with the side of the neoplasm or lobe localization (Moreale, Campanella, Marin, Skrap, & Palese, 2017). However, it is somewhat surprising that cognitive symptoms are not related to the tumor‐related characteristics. A study by Brown et al. (2004) on the prognostic effect of MMSE in patients with low‐grade gliomas compared the characteristics of glioma patients with abnormal MMSE scores and normal MMSE scores at baseline, including tumor size, tumor location, and tumor type. This study showed that there was no statistical difference in these tumor characteristics between normal and abnormal MMSE groups, which was consistent with our findings. However, it does not rule out the statistical bias caused by the small sample size and the lack of stricter grouping. In the future, we need to increase the sample size for further and more in‐depth research. Then, we found that marriage had a protective effect on the mood of glioma patients and that married people were less likely to have depressive symptoms than unmarried or divorced people were. In the face of stressful events, single people are more likely to experience a sense of helplessness due to greater stress and less social support, meaning that they are more likely to suffer from depression. Further, the KPS represents the physical function of the patient, and our study found that people who were in better health at the time of admission were more likely to feel anxious, while those who had been in poorer health were less likely to feel anxious. This may be due to the “failure of expectations” (Ekman, Wolf, Vaughan, Bosworth, & Granger, 2017).

In the next multivariate analysis, results revealed that IFN‐γ positively correlated with depressive symptoms. Precisely, glioma patients with higher levels of IFN‐γ were more likely to feel depressed. Tsao et al. have reported that elevated levels of IFN‐γ may be associated with major depressive disorder (MDD) and that the enzyme “indoleamine 2,3‐dioxygenase (IDO)” plays a role in a depression‐related signaling cascade (Gabbay et al., 2009; Simon et al., 2008; Tsao, Lin, Chen, Bai, & Wu, 2006). Induced by inflammatory stimuli, IDO breaks down tryptophan into kynurenine acid, which is then converted to serotonin (5‐HT, a neuroactive metabolite). It is well known that depression is associated with low 5‐HT function, while IDO is mainly activated by IFN‐γ, suggesting that the activation of IDO may be associated with depression (Brundin, Erhardt, Bryleva, Achtyes, & Postolache, 2015; Courtet et al., 2016; Miná et al., 2015). Compared with IFN‐γ, IL‐6, CRP, or other circulating markers of inflammation were not found to be associated with depressive symptoms in patients with gliomas. A recent meta‐analysis showed a reliable association between inflammatory markers (including CRP, IL‐1, and IL‐6) and self‐reported depressive symptoms, although studies on cancer patients remained extremely limited (Howren, Lamkin, & Suls, 2009). According to recent studies, the correlation between inflammatory factors and depressive symptoms is inconsistent across different cancers. For example, in the context of lung cancer, elevated CRP levels are associated with depression in patients with metastatic lung cancer (Mcfarland, Shaffer, Breitbart, Rosenfeld, & Miller, 2018). Conversely, in the context of breast cancer, CRP was not found associated with depressive symptoms (Bower et al., 2011). If the mechanisms of depressive symptoms vary across different cancers, this may explain why only IFN‐γ is associated with depressive symptoms in the context of gliomas. Of course, we cannot rule out that the study's sample size is small and the experimental results are biased. Therefore, we will expand the sample size in a future study.

Our results showed that IL‐2 was negatively associated with anxiety symptoms and positively correlated with cognitive impairment. There are studies that support our findings that in some animal models and clinical trials, the higher the level of IL‐2, the lower the level of anxiety (Munitz et al., 2007). And in animal experiments by Petitto, McNamara, Gendreau, Huang, and Jackson (1999), IL‐2 was shown to be a marker of systemic inflammation and was associated with decreased memory and language ability. Interestingly, many studies support the results that IL‐2 is positively correlated with anxiety (Mann, Dail, & Bailey, 2016). The researchers supposed that emotional changes were associated with fluid overload or brain edema caused by increased IL‐2 in the brain (Mann et al., 2016). However, IL‐2 is not only a pro‐inflammatory cytokine, but also an important cytokine of antitumor immunity in glioma (Rosenberg, Lotze, & Mulé, 1988). The antitumor effect of IL‐2 may affect emotion by improving the brain environment, such as reducing brain edema, which needs further study.

There are some limitations to our study: (a) The sample size of this study was relatively small and could have allowed for some bias skewing the results, (b) this study was a single‐centered and single‐person experiment, (c) this study did not compare behavioral symptoms before and after surgery, (d) no further research mechanism has been highlighted in this study, and (e) since many of the cytokines measured herein are responsive to many factors such as acute stress, and physical strain, but our study did not rule out the effects of these factors on cytokines, these may have skewed the results of this study. Furthermore, this study was limited in that only inflammatory factors in a patient's plasma were assessed, though many factors in the body can affect inflammatory factors. In addition, other possible variables, such as social support, smoking, drinking, and family burden, were ignored and not measured.

In conclusion, behavioral symptoms are prevalent in glioma patients prior to surgery, and behavioral symptoms are associated with inflammatory factors such as IFN‐γ and IL‐2. This provides further evidence of a contribution of inflammatory markers to psychological symptoms in the context of physical conditions and lays the foundation for further development of treatments for the behavioral symptoms in glioma patients. In future, we intend to use a larger number of participants, conduct multicenter research, and conduct animal experiments to explore further the underlying mechanisms. We hope that this pioneering work will inspire researchers in this field to conduct comprehensive research in this direction.

## CONFLICT OF INTEREST

We have no conflicts of interest to declare.

## AUTHOR CONTRIBUTIONS

Li Song and Xingyun Quan performed the data collection, and wrote and revised the manuscript. Li Song and Xingyun Quan are cofirst authors. Ligang Chen designed the study. Jie Zhou contributed to the conception and revision of the manuscript. Ligang Chen and Jie Zhou are co‐correspondence authors. Lin Su contributed to the conception and design of study. Ke Wang and Haorun Wang contributed to perform the analysis with constructive discussions. Lihong Wu, Chaoyi Chen, Shenjie Li, and Wei Xiang performed the data collection and analysis.

### Peer Review

The peer review history for this article is available at https://publons.com/publon/10.1002/brb3.1771.

## Supporting information

Supplementary Material

## Data Availability

The datasets used during this study are available from the corresponding author on reasonable request.
