# Supplementary material for: Inflammation and behavioral symptoms in preoperational glioma patients: Is depression, anxiety, and cognitive impairment related to markers of systemic inflammation?
Source: Brain Behav. 2020 Aug 13;10(9):e01771. doi: 10.1002/brb3.1771 (PMC7507433; doi:10.1002/brb3.1771)
Supplement: Supplementary file 1 — Supplementary Material [file BRB3-10-e01771-s001.docx]

Supplemental fig 1. Flowchart of patient eligibility, recruitment, and drop out.

New suspected gliomas (n=143)

Excluded (n = 32)

Dysphasia (n = 5)

Non-Chinese (n = 1)

Cognitive (n = 7)

Blind (n = 2)

Infection (n = 9)

Other diseases (n = 8)

Eligible

(n = 111)

Too distressed (n = 2)

Missed (n = 4)

Approached

(n = 105)

Refused (n = 9)

Consented

(n = 96)

Refuse surgery (n =16)

Do surgery, have pathological examination results

(n = 80)

Non-glioma patients (n = 9)

New gliomas

(n = 71)

Supplemental table 1. Scores of behavioral symptoms.

|  | HAMD | HAMA | MMSE |
| --- | --- | --- | --- |
| All average score | 10.3 | 10.7 | 25.4 |
| Male average score | 9.1 | 11.3 | 26.1 |
| Female average score | 11.9 | 10.0 | 24.6 |

**Supplemental material**

**Recruitment and attrition**

At the beginning of the study, 143 new patients suspected of glioma by an imaging doctor and two clinical doctors were brought to the research team, after which 111 patients were found eligible to participate. Two eligible patients were excluded. One patient was in intense pain, and we were advised by the clinician against approaching the patient. Four patients were lost to the study due to a programming error. Of the 105 patients, 96 consented to join our study. However, 16 hospitalized patients, in whom glioma was suspected, decided to exempt from surgery after the hospitalization. Nine patients subsequently dropped out of the study after the surgery because pathological findings revealed the absence of a glioma. Therefore, the final sample available for analysis consisted of 71 patients (49.7% of all hospitalized patients with suspected glioma).
